# Case report: 38-year-old female patient with severe legionellosis but no source of infection

**DOI:** 10.3205/dgkh000329

**Published:** 2019-10-17

**Authors:** Benedikt M. J. Lampl, Werner Strigl, Dieter Winner, Michael T. Pawlik

**Affiliations:** 1 Public Health Department Regensburg, Germany; 2 Caritas-Krankenhaus St. Josef, Department of Anesthesiology, Regensburg, Germany

**Keywords:** legionellosis, risk factors, infection prevention, exposure, ECMO

## Abstract

We report about a 38-year-old female patient with an extremely severe case of legionellosis. The patient had to be treated in the intensive care unit for more than a month. Extracorporeal membrane oxygenation (ECMO) had to be established in order to save the patient’s life. The patient did not belong to any risk group (i.e., age >50, smoker, immunosuppression, chronic disease, male sex). Infection control investigations carried out by the Public Health Department could not reveal concrete exposures likely to cause the infection. The serotypes in the patient material (*Legionella pneumophila* serotype 2–14) and in the samples taken from the showerhead in the patient’s apartment (*Legionella pneumophila* serotype 1) were not identical. Results of the examination performed according to the German Drinking Water Ordinance (Trinkwasserverordnung, TrinkwV) carried out in 2017 in the patient’s apartment, showed that the technical measures limit (Technischer Maßnahmenwert, TMW) was not exceeded. The patient has survived through the extensive use and efforts of intensive care measures. In general, a concrete exposure to Legionella is often not ascertainable, as in the case presented. This raises the question of how and whether such cases of legionellosis are preventable.

## Zusammenfassung

Wir berichten über eine 38-jährige Patientin mit schwerstem Verlauf einer Legionellose. Die Patientin musste länger als einen Monat auf der Intensivstation behandelt werden. Eine Therapie mit extrakorporaler Membranoxygenierung (ECMO) war notwendig, um das Leben der Patientin zu retten. Die Patientin gehörte keiner Risikogruppe an (d.h. Alter >50, Raucher, Immunsuppression, chronische Erkrankung, männliches Geschlecht). Ermittlungen des Gesundheitsamtes im Rahmen des Infektionsschutzes erbrachten keine konkrete ursächliche Exposition. Die Serotypen im Patientenmaterial (*Legionella pneumophila *Serotyp 2–14) und in den aus dem Duschkopf in der Patientenwohnung entnommenen Proben (*Legionella pneumophila* Serotyp 1) waren nicht identisch. Bei der 2017 in der Patientenwohnung durchgeführten Untersuchung gemäß Trinkwasserverordnung (TrinkwV) wurde keine Überschreitung des Technischen Maßnahmenwertes (TMW) festgestellt. Die Patientin hat durch Einsatz hochkomplexer intensivmedizinischer Maßnahmen überlebt. Häufig ist eine konkrete Exposition gegenüber Legionellen, wie im vorliegenden Fall, nicht feststellbar. Das wirft die Frage auf, wie und ob solche Fälle von Legionellose vermeidbar sind.

## Introduction

Legionella are gram-negative aerobic bacteria found in aquatic habitats throughout the world [1]. Legionella proliferate intracellularly in amoebae or other protozoa; optimal temperatures for their growth are between 25°C and 45°C. The most relevant species for humans is *Legionella pneumophila* with serogroup 1, which causes about 90% of the diseases [1]. Risk factors are immunosuppression, alcohol and tobacco abuse, age >40–50 years and male sex [2]. The inhalation of aerosols containing Legionella, e.g., in the domestic shower, is regarded the main route of transmission [1]. Legionella can cause two diseases: Legionellosis, also called Legionnaire’s disease, and Pontiac fever. Legionellosis is often a severe disease with a fatality rate of 10% to 15%. In Germany, an annual number of legionellosis cases of about 15,000 to 30,000 is assumed [1], [3]. In order to reduce the risk of infection by Legionella in drinking water, the Drinking Water Ordinance [4] requires routine testing every three years and the initiation of measures with proof of a concentration of more than 100 CFU/100 ml in a certain pipeline volume in commercial properties (and therefore also in rental apartments).

## Clinical Course

A 38-year-old, slightly obese patient (BMI 28), who worked as a room cleaner in a children’s home and was healthy with an inconspicuous general medical history, had noticed a deterioration of her general condition with exhaustion, increased body temperature, and shortness of breath since 16/01/2019. She went to her family doctor and was treated under the suspicion of a viral infection of the upper respiratory tract. On 21/01/2019 the symptoms rapidly worsened and the patient presented to the interdisciplinary emergency room with a septic general condition, tachypnea and decreased gas exchange. Rattling noises of the left lower and middle field of the lungs were noticeable, the chest X-ray showed infiltrates.

The patient was transferred (under treatment with ampicillin/sulbactam started by the family doctor) from the normal ward to the intensive care unit for non-invasive ventilation (NIV) after only 6 hours. With 10 liters of oxygen/min and a respiratory rate of 35/min, an oxygen saturation of only 89% could be achieved via mask. Under NIV, a respiratory alkalosis with pH 7.55 was observed with persistent dyspnea. For better tolerance, NIV was alternately combined with high-flow therapy. Antibiotic therapy was continued and supplemented with clarithromycin. A rapid influenza test and a rapid Legionella test in urine were negative on the same day. 

On 22/01/2019, NIV therapy showed an increasingly poor gas exchange with tachypnea, so the patient had to be intubated due to respiratory exhaustion. Afterwards, bronchoscopy with BAL was performed. The patient was placed on her abdomen to improve gas exchange, which was still insufficient. The antibiotic regimen was intensified by piperacillin/tazobactam and continued for 7 days. The positioning therapy did not yield sufficient success. As the pulmonary situation worsened increasingly, the indication for venovenous extracorporeal membrane oxygenation (vvECMO) emerged, which was started on 23/01/2019 without complications by inserting two cannulae into the right femoral and jugular vein. Immediately after beginning vvECMO, gas exchange improved and the patient was first ventilated “ultraprotectively” with respiratory volumes of 3 ml/kg body weight.

On the same day, a further de-escalation of the ventilation strategy was possible by switching to CPAP ventilation. At the same time, *Legionella pneumophila* was detected in the BAL, so Levofloxacin was added. Gas exchange stabilized under vvECMO and pulmonary edema decreased with a negative fluid balance. For anticoagulation, Heparin was administered with a target PTT of 70–80 sec. With significantly increased fibrinogen values of 550 mg/dl, additional epoprostenol was administered as “thrombocyte coating” via iLA (interventional lung assist) ahead of the oxygenation filter. 

After one week of vvECMO therapy, however, the pulmonary situation did not improve significantly, thus computer tomography of the skull, thorax and abdomen was performed. The lungs continued to be the focus of sepsis with severe ARDS. In the prone position, the patient developed supraventricular tachycardia which had to be cardioverted due to the compromised hemodynamics. As a result, gas exchange initially improved considerably. The need for catecholamine increased drastically; the patient received additional volume and several transfusions due to a significant drop in hemoglobin. With the patient becoming increasingly hemodynamicly instable, FAST sonography showed free fluid in the liver bed. The patient was immediately subjected to an exploratory laparotomy. During the procedure, a hemorrhage in the left rectus abdominis muscle with intramuscular hematoma was observed, which had spread intraabdominally and retroperitoneally. Perioperatively, a massive transfusion was necessary with an estimated blood loss of 4000 ml. The need for catecholamine decreased rapidly after surgery, but further transfusions were necessary. Immediately after surgery, continuous venovenous hemofiltration (CVVH) was started because of acute renal failure. During the further course, relaparotomy was necessary due to recurrent hemorrhaging of the rectus abdominis muscle. During the operation, 1.5 liters of old blood and 3 liters of free fluid were removed from the abdominal cavity.

The abdominal pressure relief improved the hemodynamics so that the catecholamine doses could be halved. ECMO therapy now proceeded without disturbances and with reduced blood flow. Catecholamines could be reduced stepwise. After removal of the abdominal swabs, the positioning therapy had to be continued because of continuing bilateral atelectases. In the further course, gas exchange improved considerably. The blood flow could be reduced under adapted ventilation with higher tidal volumes. Sedation could be gradually reduced and weaning from the ventilator was initiated. 

On 07/02/2019, CVVH was stopped and negative balances could be achieved with the administration of loop diuretics. The patient became more alert and breathed spontaneously with pressure support. On 09/02/2019, the intraperitoneal Robinson drain was again bloody with a drop in hemoglobin. A CT scan of the thorax and abdomen revealed an active muscular hemorrhage of the left rectus abdominis with a hematoma in the left middle abdomen. Anticoagulation with heparin was adjusted to a target PTT of 40–45 sec, after which there was no further signs of active bleeding. On 11/02/2019, ECMO therapy could be terminated, as gas exchange was sufficient. Sedation was reduced and the patient became awake and responsive without neurological deficiencies. Because of an infected intra-abdominal hematoma, surgery was necessary again on 13/02/2019. The patient was extubated without any problems on 15/02/2019.

With increasing infection parameters, bilious reflux, pressure pain and a three-layered gall bladder shown by ultrasonography, a calculated antibiotic therapy was started. Nonetheless, the patient developed fever continua, sinus tachycardia, increasing bilirubin and infection parameters. Thus the indication for cholecystectomy was made and a gangrenous gall bladder was removed. In the further course, the oral food intake was increased stepwise and gastrointestinal function normalized with declining liver values.

On 21/02/2019, 36 days after admission to the intensive care unit, the patient was finally transferred to the intermediate care unit. After 4 days, the patient was transferred to the normal ward, from where she was discharged 10 days later for subsequent rehabilitation treatment.

## Infection control investigations

On 07/02/2019, a positive result for Legionella in bacteriological culture and a positive PCR result for *Legionella pneumophila*/Legionella spp. from the bronchoalveolar lavage (BAL) of a 38-year-old female patient was reported to the Public Health Department. The investigations, including interviews with the patient’s husband and employer, did not reveal any evidence of a specific occupational or private exposure (e.g., use of showers in a sports club, sauna etc.) to Legionella. According to her husband, the patient had only used the domestic shower within the incubation period of the disease. A drinking water sample from the building of the patient’s apartment obtained in July 2017 showed that the TMW had not been exceeded. The patient was a member of the cleaning staff in a children’s home. From this institution, results of Legionella testing from July 2018 complied with the requirements of the German Drinking Water Ordinance (Trinkwasserverordnung) and did not exceed the TMW. 

Samples from the drinking water system (type “e” for so-called purpose “c” [5]) were taken in the patient’s apartment by the Public Health Department. The showerhead and sink were sampled. In the showerhead, Legionella were detected with 100 CFU/100ml. The laboratory diagnostics revealed *Legionella pneumophila* serogroup 1. In the patient’s BAL, *Legionella pneumophila* serogroup 2–14 was determined. 

Further investigations did not identify any potential exposure in the patient’s working environment. Although the patient worked as a cleaner and was also involved in the cleaning of sanitary areas, situations in which the patient was likely to be significantly exposed to (sufficient) water vapor/aerosols and thus potentially to Legionella were not detected. Ultimately, a causal exposure could not be determined. Other cases of legionellosis in the patient’s private or professional environment did not occur.

## Discussion

The patient’s case impressively proves that Legionella can cause the most serious pulmonary infections [6]. It is puzzling, though, why a young and otherwise healthy person became infected with Legionella. This case is exceptional in that the patient had none of the typical risk factors (age >50, smoker, immunosuppression, chronic disease, male sex) [2]. From a clinical or intensive care medical point of view, it is remarkable that the patient survived the severe ARDS with a need for extracorporeal oxygenation after a fulminant course and considerable complications (life-threatening hemorrhages, cardiac arrhythmias, acute renal failure, multiple laparotomies, cholecystectomy). In addition, the patient’s case illustrates a common issue for those dealing with infection control in public health service: exposure categories such as “private”, “occupational”, or “travel” are identifiable, but exposures are often not accurately assignable temporally and spatially. A causal connection of Legionella in the plumbing system, for example, and legionellosis in a certain patient often cannot be established. In this case too, the serotypes in the patient material and in the samples taken from the showerhead were not identical. Other concrete exposures likely to cause the infection were not detectable. The results of the routine testing in the patient’s apartment in 2017 showed that the technical measures limit (TMW) had not been exceeded. 

The general question that arises is whether and to what extent the regular mandatory monitoring of Legionella according to the Drinking Water Ordinance (≤100 CFU/ 100ml, one examination every three years in apartment buildings, if limit not exceeded) is suitable for preventing legionellosis and its most severe course as in the patient described. In this case, the test results from the year 2017 were unremarkable, but the Legionella in the c-sample of the domestic shower hose did not cause the infection. Apart from this, the risk of infection with proof of Legionella does not increase linearly with concentration, as there is a paradoxical dose-response relationship due to Legionella being more contagious when amoebas are present [1]. Moreover, the role of VBNC (viable but non-culturable) forms of Legionella in the risk of infection is unclear [7]. Hence, the relevance of a certain Legionella concentration for infection remains vague, even if detectable.

## Conclusions

In individual cases, Legionella can cause the very severe disease even in patients with a low risk profile. A concrete exposure event is often not ascertainable, as in the case presented. The routine testing of the drinking water of the house plumbing according to the German Drinking Water Ordinance was not suitable to prevent the infection. Investigations concerning adequate exposures to Legionella do often not yield results. In this case, the patient has survived through the extensive use and efforts of intensive care measures and – presumably – because she was young and otherwise healthy. All possible efforts should be made to investigate the details of transmission, virulence factors, and epidemiology of legionellosis and to evaluate the effectiveness of measures for preventing this disease.

## Notes

### Competing interests

The authors declare that they have no competing interests.
